# Optimizing the Flavor Profile of Brazilian Spirits: Torrefaction Modeling of Native Woods for *Cachaça* Maturation

**DOI:** 10.3390/molecules31040633

**Published:** 2026-02-12

**Authors:** Amanda F. Reitenbach, Adriana Sturion Lorenzi, Nicole P. Catibe, Renata P. I. Tormena, Diego C. B. D. Santos, Ana Carolina Broch, Edgar A. Silveira, Talita Souza Carmo, Paulo Anselmo Z. Suarez, Grace F. Ghesti

**Affiliations:** 1 Laboratory of Bioprocesses, Materials and Fuels, Institute of Chemistry, University of Brasília, Brasilia 70904-970, DF, Brazil; 2 Science of Beer Research Group, Science Inovatech Institute, Florianopolis 88020-000, SC, Brazil; 3 Energy Environment Laboratory, Institute of Chemistry, University of Brasília, Brasilia 70904-970, DF, Brazil

**Keywords:** Brazilian woods, wood toasting, maturation, *cachaça*, Brazilian distilled spirit, artificial intelligence

## Abstract

*Cachaça*, a traditional Brazilian spirit, undergoes significant sensory refinement through barrel aging. In this study, we investigated how heat treatment of Brazilian woods (Balsam, Jaqueira, Jequitibá, Amburana, and Ipê) affects the sensory profile of *cachaça*, using Oak as a benchmark. Physicochemical characterization, toasting assessments, sensory analysis, and artificial intelligence (AI) were integrated to develop a predictive model for optimizing wood selection and heat-treatment conditions to achieve targeted sensory profiles. Applying this model, we produced a five-wood *cachaça*, a novel spirit distinguished by its complexity and customized sensory attributes. This approach reveals that each wood species develops distinct characteristics depending on toasting parameters such as time and temperature, challenging the current Brazilian practice where a single toasting condition is applied to all woods without prior physicochemical analysis. Linking wood composition with sensory outcomes through AI, this work introduces an unprecedented product innovation and demonstrates the potential of multi-criteria analysis to guide spirit maturation, enhance product design, and reshape the beverage industry.

## 1. Introduction

Distilled spirits frequently display harsh, bitter, or astringent sensory attributes immediately after production, which may limit consumer appreciation of their complexity. Wood maturation represents a critical step in distilled beverage production, during which chemical interactions between the wood matrix and the spirit play a central role in the development of color, aroma, and flavor (Silvello et al., 2021; Conner, 2022; Tarko et al., 2023) [1,2,3]. These transformations arise primarily from the thermal degradation of wood biopolymers (e.g., cellulose, hemicellulose, and lignin) during seasoning and toasting, followed by the extraction and further evolution of their degradation products during aging (Angeloni, 2015; Wyler et al., 2015; Bortoletto et al., 2016; Silveira et al., 2023) [4,5,6,7]. Compounds such as phenolic aldehydes, lactones, furanic derivatives, and volatile phenols contribute directly to sensory attributes (Silvello et al., 2021) [1], including sweetness, vanilla-like notes, smokiness, and astringency.

In particular, maturation in wooden barrels has been shown to markedly improve sensory quality by promoting balance and enhancing both depth and complexity (Silvello et al., 2021) [1]. A schematic representation of spirit maturation using reused barrels, virgin barrels, or the addition of wood pieces to impart flavor, constructed from literature-derived data, is presented in Supplementary Figure S1. In the context of *cachaça*, the use of native Brazilian wood species represents both a cultural tradition and a technological opportunity for sensory diversification. Unlike oak (*Quercus* spp.), whose chemical behavior during thermal treatment and aging has been extensively characterized (Bortoletto et al., 2016; Silvello et al., 2021) [1,6], many Brazilian woods exhibit distinct lignin compositions, extractive profiles, and thermal response patterns. These differences suggest the potential for unique sensory signatures, but also introduce substantial complexity in process control, particularly when toasting conditions are empirically defined.

Previous studies have described the chemical composition of toasted woods and their effects on aged *cachaça*. However, most investigations remain largely descriptive, focusing on isolated wood species, single toasting intensities, or post-aging chemical profiles (Bortoletto et al., 2016; Silvello et al., 2021) [1,6]. While these studies provide valuable qualitative insights, they rarely establish systematic relationships between controllable process parameters such as temperature and time, wood composition, and resulting sensory perception. Consequently, producers continue to rely heavily on trial-and-error approaches, limiting reproducibility and scalability (Holds et al., 2025) [8].

At the molecular level, thermal treatment governs the balance between desirable and undesirable sensory contributors. Moderate toasting promotes the formation of low-molecular-weight phenolics and furanic compounds associated with sweetness and aromatic complexity, whereas excessive toasting may lead to the degradation or volatilization of key odorants and the formation of harsh, smoky, or bitter notes (Proskurina et al., 2017; Wei-Hsin et al., 2021; Silveira et al., 2023) [7,9,10]. Despite this well-recognized trade-off, few studies explicitly integrate molecular composition, sensory outcomes, and processing parameters into a unified analytical framework.

Wood composition is a critical determinant of flavor development, consisting primarily of holocellulose (≈70%), lignin (≈25%), and extractives (≈4%). Holocellulose comprises cellulose and hemicellulose, which contribute differently to sensory perception (Supplementary Figure S2). Cellulose has a limited direct impact on flavor, whereas hemicellulose contributes to mouthfeel and caramelized sensory notes. The thermal degradation of lignin yields aromatic compounds commonly associated with vanilla-like characteristics, while extractives—particularly tannins—contribute to astringency and assist in the removal of undesirable volatile compounds (Leão et al., 2006) [11]. Drawing on literature-derived data, Supplementary Table S1 presents key wood-derived compounds involved in maturation and their contributions to sensory characteristics. Recent advances in data-driven modeling have been suggested to address this gap by enabling the exploratory prediction of sensory trends from experimental descriptors, in line with the multivariate and machine-learning approaches reported by Silveira et al. (2021) [7]. In this context, predictive models are not intended to replace chemical or sensory analyses, but rather to support decision-making by identifying process regions associated with favorable sensory responses. When applied cautiously, such models can reduce experimental space and guide rational process design, particularly in complex systems involving multiple wood species and thermal regimes.

Therefore, the objective of this study was to evaluate the effects of controlled toasting conditions applied to selected Brazilian wood species on physicochemical characteristics and sensory perception during *cachaça* maturation, and to integrate these variables into a predictive modeling framework. We hypothesized that temperature–time combinations could be systematically associated with sensory tendencies through compositional descriptors, enabling the identification of toasting conditions that balance aromatic complexity and sensory harmony. Through the integration of experimental data with predictive analysis, this study aims to contribute a structured and reproducible approach to the rational use of Brazilian woods in *cachaça* aging, as an alternative to the commonly used oak.

## 2. Results

### 2.1. Wood Analysis

The Brazilian wood species evaluated were Amburana (*Amburana cearensis*), Balsam (*Myroxylon balsamum*), Ipê (*Tabebuia* spp.), Jequitibá (*Cariniana* spp.), and Jaqueira (*Artocarpus heterophyllus*), with Oak (*Quercus* spp.) included as a reference material. The results of their composition analyses are summarized in Table 1 and Table 2. Table 1 presents the extractives and moisture contents, while Table 2 reports ash (without extractives), lignin, and holocellulose contents.

According to Table 1, the woods with the highest extractive contents were Balsam (16.57%), Oak (16.55%), and particularly Ipê (28.19%), indicating elevated levels of volatile and aromatic compounds capable of influencing *cachaça* maturation. Among the samples, Ipê exhibited the highest extractives content (28.19%), whereas Jaqueira displayed the lowest moisture content (4.34%).

Balsam (0.75%), Amburana (0.84%), and Jequitibá (0.62%) exhibited the highest ash contents after extractive removal, indicating elevated levels of inorganic constituents such as calcium, magnesium, and zinc (Table 2). These minerals may influence the colloidal stability of the matured beverages and, consequently, affect their clarity and sensory stability.

The samples with the highest lignin contents were Jaqueira (50.14%) and Ipê (46.89%), followed by Amburana (43.78%) and Oak (40.39%) (Table 2). These results indicate that different conditions of heat treatment can influence lignin concentration, likely due to the preferential degradation of cellulose and hemicellulose during toasting. The elevated lignin content observed in Jaqueira also suggests greater wood hardness and structural resistance.

**Figure 1:**
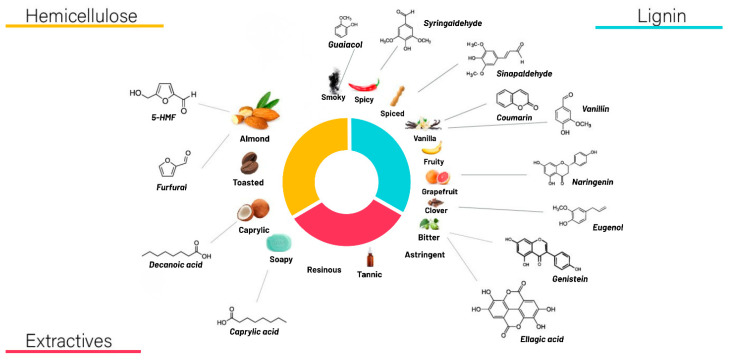
Chemical constituents of wood and their associated sensory profiles, adapted from Guimarães et al., 2020 [12], and applied in the present study.

With respect to holocellulose composition, Oak (59.53%), Jequitibá (56.26%), and Amburana (56.07%) presented the highest proportions, reflecting an inverse relationship with their lignin levels (Table 2). As holocellulose undergoes thermal degradation during heat treatment, the resulting increase in exposed lignin surface area favors the extraction of aromatic constituents and other flavor-active compounds produced from both lignin depolymerization and carbohydrate decomposition (Figure 1).

Amburana exhibited yellowish-beige tones, whereas Balsam showed coloration ranging from dark to reddish brown. Ipê presented a dark yellowish-brown appearance, while Jequitibá was characterized by a light coloration. Jaqueira displayed a distinctive yellowish hue.

### 2.2. Treatment Ranking and Sensory Analysis

The sensory analyses were conducted at the University of Brasília (UnB), where a panel of judges was established through structured theoretical and practical training sessions using an off-flavor reference kit (FlavorActiv). The training aimed to qualify panelists and to consolidate a sensory evaluation group within UnB. This panel was responsible for the assessment of all samples investigated in this study. Panelists evaluated three or more coded samples per session and ranked them in descending order of preference. Among the seven assessors, the greatest variability in preference was associated with roasting time, whereas roasting temperature elicited unanimous preferences for specific batches. Sensory results were organized into two attribute groups (aroma and flavor) and are presented as spider plots in the Supplementary Figure S3. The principal observations derived from these analyses are summarized in Table 3.

### 2.3. Predictive Model Proposal

The sensory results, combined with the compositional attributes of each wood species and the desired flavor profile (Figure 1), physicochemical data, roasting intensity, and literature evidence, served as the basis for building an AI-assisted predictive model for wood roasting in *cachaça* maturation. Accordingly, this information was provided to Gemini 3 Flash to generate a model based on the descriptors reported in the sensory analysis (Table 4).

Based on this recommendation, the five-wood species were selected according to their national availability and commercial supply for barrel production, in which the spirits were aged individually. Each spirit was matured for one year under the optimized conditions and subsequently blended in equal proportions to obtain the final product, referred to as the five-wood *cachaça*.

**Figure 2:**
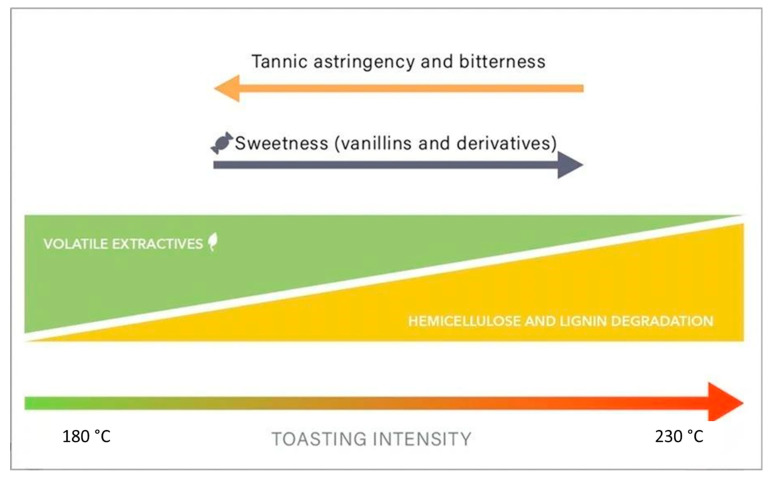
Simplified model, generated in the present study, depicting how increasing toasting temperature modulates astringency, bitterness, and sweetness.

Figure 2 demonstrates that increasing toasting intensity favors the loss of astringent compounds and enhances the thermal breakdown of hemicellulose and lignin, leading to the generation of aroma-active compounds that improve sweetness, attenuate bitterness, and increase sensory complexity.

According to this model, toasting intensity plays a central role in defining the chemical composition and sensory attributes of aged spirits. At lower toasting temperatures (approximately 80 °C), the wood retains a higher concentration of volatile extractives, resulting in pronounced tannic astringency and bitterness in the final beverage. As toasting temperatures approach 200 °C, the degradation of hemicellulose and lignin intensifies, favoring the release of compounds that enhance sweetness and reduce bitterness and astringency. The balance between these contrasting attributes is fundamental for achieving the desired flavor profile.

These findings highlight the potential of integrating multi-criteria analysis with artificial intelligence to optimize aging conditions and broaden the range of flavor profiles available to the beverage industry. In this context, the proposed model enables precise modulation of sensory attributes, facilitating the rational design of complex and customized flavor profiles, as exemplified by the development of the five-wood *cachaça*. Evaluation of the AI-assisted predictive model demonstrated satisfactory statistical performance, with ANOVA results (Table 5) showing a calculated F value exceeding the tabulated threshold and explaining approximately 67.4% of the total variance. These findings demonstrate that the model accurately represents the effects of toasting temperature and duration on wood properties and associated sensory attributes, thereby supporting its applicability to flavor optimization in *cachaça* production.

### 2.4. Development and Physicochemical Analysis of the Final Five-Wood Cachaça

Following the optimization of wood-toasting conditions guided by the AI-assisted predictive model, the distillates from each wood species were matured and subsequently blended in equal proportions to produce the five-wood *cachaça*, which was then subjected to comprehensive physicochemical characterization. Analyses confirmed the overall quality of the resulting spirit, with an alcoholic strength of 39.3 °GL, a relative density of 0.95, and a volatile acidity of 79.29 mg L^−1^. The higher alcohol profile was dominated by isoamyl (122.08 mg L^−1^), isobutyl (69.24 mg L^−1^), and n-propyl (57.23 mg L^−1^) alcohols, while total esters reached 46.51 mg L^−1^. Furfural and aldehyde concentrations were 6.13 mg L^−1^ and 14.31 mg L^−1^, respectively. Methanol and ethyl carbamate levels remained within safe regulatory limits, reflecting proper fermentation, controlled aging conditions, and effective extraction of flavor compounds (Table 6).

## 3. Discussion

Wood selection and barrel characteristics strongly influence *cachaça*’s chemical composition and sensory profile by affecting compound extraction, oxygen transfer, and microbial interactions (Bossaert et al., 2021; Junqua et al., 2021; Rowell, 2021) [13,14,15]. Evidence from craft beer, mead, and tequila shows that controlled maturation, including wood type, heat treatment, and aging conditions, modulates flavor-active compounds and enhances sensory complexity (Reitenbach et al., 2025a, 2025b, 2026; Warren-Vega et al., 2021) [16,17,18,19]. Applying these principles to *cachaça* enables the design of spirits with targeted aroma and taste profiles while maintaining consistent quality (Silvello et al., 2021; Briceno et al., 2025) [1,20].

The chemical composition of the Brazilian wood species analyzed (Amburana, Balsam, Ipê, Jequitibá, and Jaqueira) revealed marked differences in extractives, lignin, holocellulose, and mineral content, which are key determinants of flavor development during spirit aging (Leão, 2006; Tarko et al., 2023) [3,11]. Ipê exhibited the highest extractives content (28.19%), consistent with its potential to impart pronounced aroma and flavor characteristics due to the higher concentration of volatile compounds (Briceno et al., 2025) [20]. Balsam and Oak, with extractive contents exceeding 16%, also represent significant sources of aromatic constituents capable of influencing the sensory profile of *cachaça* (Rodrigues et al., 2016; Guimarães et al., 2020) [12,21]. Conversely, Jaqueira’s low moisture content (4.34%) suggests reduced water-mediated compound loss during heat treatment, which may influence both extraction efficiency and flavor intensity (Liang et al., 2020) [22].

The ash contents of Amburana (0.84%), Balsam (0.75%), and Jequitibá (0.62%) indicate elevated levels of inorganic constituents, such as calcium, magnesium, and zinc, which may contribute to colloidal stabilization and affect beverage clarity and mouthfeel (Da Silva et al., 2021) [23]. Lignin content varied substantially across species, with Jaqueira (50.14%) and Ipê (46.89%) displaying the highest values. This finding suggests that these woods possess greater structural resistance and potential for generating lignin-derived aroma-active compounds during thermal treatment. The inverse relationship between holocellulose and lignin content—most evident in Oak (59.53% holocellulose; 40.39% lignin)—aligns with literature reports indicating that hemicellulose and cellulose degradation during toasting exposes lignin, facilitating the formation of vanillin, syringaldehyde, and related flavor compounds (Leão, 2006; Guimarães et al., 2020) [11,12].

Sensory analysis corroborated that wood species and maturation practices influenced flavor intensity and complexity, with differences in chemical composition likely contributing to these effects (Bortoletto et al., 2016; Silvello et al., 2021) [1,6]. Amburana, characterized by highly spiced aromas and flavors, consistently exhibited the highest sensory intensities, while Ipê and Oak produced complex flavor profiles combining resinous, vanilla, and spiced notes (Wyler et al., 2015; Rodrigues et al., 2016) [5,21]. Jequitibá and Balsam contributed more specific aromas, such as vanilla and malt notes, which reflect the selective extraction of lignin- and carbohydrate-derived compounds during controlled heat treatment (Leão, 2006; Tarko et al., 2023) [3,11]. Notably, the panel responses highlighted the strong influence of roasting time and temperature on flavor perception, confirming that both factors are critical levers for modulating astringency, bitterness, and sweetness (Guimarães et al., 2020; Briceno et al., 2025) [12,20].

In line with recent approaches (Silvello et al., 2021; Warren-Vega et al., 2023) [1,24], the predictive model developed in this study integrates wood composition, thermal treatment, and sensory data to propose optimized toasting conditions for each species, supporting targeted modulation of *cachaça* flavor profiles. The model (Figure 2) proposes a mechanistic interpretation in which lower toasting temperatures preserve extractives, producing more pronounced tannic astringency, whereas higher temperatures (~200–230 °C) favor hemicellulose and lignin degradation, enhancing sweetness and attenuating bitterness (Leão, 2006; Guimarães et al., 2020) [11,12]. ANOVA indicated a statistically significant regression, with an F-value exceeding the critical threshold and 67.4% of the variance explained, reflecting moderate explanatory power that is commonly considered acceptable in sensory science, given the inherently high biological and perceptual variability of sensory data (Warren-Vega et al., 2023) [24].

Implementation of the model guided the production of the five-wood *cachaça*, which demonstrated balanced physicochemical and sensory characteristics (Santiago et al., 2024) [25]. The final spirit exhibited appropriate alcoholic strength (39.3 °GL), controlled volatile acidity (79.29 mg L^−1^), and a higher alcohol profile dominated by isoamyl, isobutyl, and n-propyl alcohols, reflecting proper fermentation and effective extraction from the selected woods (Alcarde et al., 2014; Bortoletto et al., 2016) [6,26]. Importantly, methanol and ethyl carbamate remained below regulatory limits, ensuring the safety and quality of the product (Da Silva et al., 2021) [23]. The blending of individually aged spirits under optimized conditions produced a complex flavor profile, indicating that multi-wood aging, guided by AI-assisted predictive modeling, can broaden the sensory repertoire of *cachaça* while maintaining regulatory compliance and physicochemical integrity (Silvello et al., 2021; Warren-Vega et al., 2023) [1,24].

Regulatory frameworks for spirits primarily focus on aging conditions, vessel type, minimum maturation periods, and labeling requirements, rather than specifying exact toasting temperatures or durations (Conner, 2022) [2]. In Brazil, *cachaça* producers must comply with standards set by MAPA regarding barrel use and aging practices to ensure product identity and quality (Bortoletto et al., 2016) [6]. Internationally, regulations such as the U.S. TTB rules and EU Regulation 2019/787 define spirit classifications based on aging in specific wood types (e.g., charred oak for whiskey) and minimum maturation periods (Conner, 2022) [2]. While these frameworks influence how barrels are prepared, specific toasting or charring protocols remain largely industry practices aimed at achieving desired sensory profiles rather than legal mandates (Tamayo-Sánchez et al., 2023) [27].

Although the results obtained are promising, some limitations should be considered. The study examined a limited number of Brazilian wood species in comparison with oak, and the aging period was restricted to one year, which may not fully reflect the dynamics of long-term maturation (Leão, 2006) [11]. The AI-assisted predictive model was constructed using a relatively small dataset, and sensory evaluation was performed by a single trained panel, which may restrict the transferability of the findings to other production conditions or consumer populations (Silvello et al., 2021; Warren-Vega et al., 2023) [1,24]. Additionally, the experimental scope was limited to specific toasting temperatures and durations, and the complex interactions among individual wood-derived compounds during aging were not extensively investigated. Future studies expanding the range of wood species, aging times, sensory panels, and chemical analyses would contribute to improving predictive robustness and supporting the broader application of multi-wood maturation strategies in *cachaça* production.

Finally, these results highlight the synergistic potential of integrating wood compositional analysis, controlled heat treatment, sensory evaluation, and artificial intelligence in spirit maturation, reinforcing insights previously reported for predictive and data-driven approaches in distilled beverages (Silvello et al., 2021; Warren-Vega et al., 2023) [1,24]. The study also demonstrates that specific Brazilian wood species, beyond the conventional use of Oak, can be strategically employed to modulate flavor attributes, offering both artisanal and industrial applications. Moreover, this approach provides a rational framework for designing spirits with targeted sensory profiles, optimizing the extraction of desired compounds, and minimizing off-flavors, contributing to innovation in *cachaça* production and other barrel-aged spirits.

## 4. Materials and Methods

### 4.1. Wood Samples

The wood samples used in this study were acquired through the company Dornas Havana (Taiobeiras, MG, Brazil), which provided a kit with wooden dice without toast made from species from several Brazilian biomes, mainly from the Atlantic Forest and the Amazon. The kit contained approximately 100 dice of each species with dimensions of 1 cm × 1 cm. (Table 1). All wood materials were sourced from regions with the requisite environmental permits and were accompanied by a Forest Origin Document (Documento de Origem Florestal—DOF), in accordance with Ordinance No. 253 of 18 August 2006, issued by the Ministry of the Environment (MMA). The DOF constitutes a mandatory certification for the transport and storage of products derived from native forests. The wood samples were primarily sourced from reforestation plantations or areas affected by flooding due to the construction of hydroelectric dams. Following preliminary evaluations, 5 wood species were selected for experimental analysis (Table 7). Oak, the conventional wood used in spirit aging, was employed as a reference to assess the maturation potential of the selected species. The sample cubes (1 cm^3^) were subjected to a range of temperature and toasting conditions (Supplementary Table S2).

### 4.2. Wood Analysis

#### 4.2.1. Moisture, Ash and Extractable Contents

Moisture and ash contents of the wood samples were determined in triplicate to ensure analytical reproducibility. The moisture content was obtained from 1 g of milled wood sample with grain size less than 60 mesh. The samples were weighed and placed in an air circulator (TECNAL, model TE-394-1, Piracicaba, SP, Brazil) with circulation and renewal of air model 035, at 105 °C. The samples remained in the oven until the constant mass. After this period, the samples were cooled in a desiccator and after total cooling, weighed to determine the moisture content t in percentage. For the ash content test, similar samples were placed in porcelain crucibles of known mass and then placed in a mufla (FORNITEC, Sao Paulo, SP, Brazil), previously heated to 700 °C. The crucibles remained inside the mufla for 5 h until the total calcination of the material. After cooling in the dryer, the masses were weighed to calculate the percentage ash content.

Extractable content was assessed using a procedure adapted from the TAPPI T 204 om-88 standard (TAPPI, 1989) [28] of the Technical Association of the Pulp and Paper Industry, which involves solvent extraction to quantify the fraction of wood components soluble. Three round-bottom balloons were placed in an air circulator (TECNAL, model TE-394-1, Piracicaba, SP, Brazil) at 115 °C for 2 h, cooled in a desiccator, weighed on an analytical balance (Mettler, model AE 160, Zevenhuizen, Netherlands), and then 210 mL of toluene solvent: ethanol (1:2) was added. In a cellulose cocoon, 2 g of wood with granulometry in the range of 60–100 mesh were weighed to perform extraction by Soxhlet. The extraction was carried out in an average time of 6 h and the balloon was dried in the greenhouse (Marconi, model MA 037, Piracicaba, SP, Brazil), for a period of 2 h at 115 °C.

#### 4.2.2. Lignin and Holocellulose Contents

For the isolation of acid lignin, wood samples were first ground, and extractives were removed using a 1:2 ethanol–toluene solution, following the TAPPI T 204 om-88 standard (Solvent Extractives of Wood and Pulp) (TAPPI, 1989) [28]. Soluble and insoluble lignin contents were subsequently determined according to laboratory procedures LAP #003 and LAP #004, respectively (Templeton et al., 1995) [29]. Total lignin content was calculated as the sum of the insoluble and soluble fractions, using Equation (1):

Total Li = Insoluble Li + Soluble Li(1)

where Total Li represents the total lignin content, Insoluble Li corresponds to the insoluble lignin fraction, and Soluble Li corresponds to the soluble lignin fraction.

Holocellulose content, excluding extractives, was calculated by difference, subtracting total lignin and ash contents from 100%, according to Equation (2):

TH = 100% − Total Li − AC(2)

where TH denotes the total holocellulose content (%), AC represents the ash content (%), and Total Li is the total lignin content (%).

#### 4.2.3. Wood Toasting System

The thermal treatment of wood samples was conducted using a MACRO TGA-2000 thermogravimetric analyzer (Navas Instruments, Conway, SC, USA). The experimental setup (Supplementary Figure S4) comprised a nitrogen (N_2_) cylinder, a gas control rotameter, the thermogravimetric unit for sample loading, and a computer for data acquisition and subsequent statistical analysis. The resulting dataset supported the development of an artificial intelligence–based model designed to create next-generation matured beverages with optimized chemical and sensory profiles.

## 5. Experimental Procedures

Wood samples were subjected to thermogravimetric analysis (TGA) using a MACRO TGA-2000 system equipped with a carousel capable of accommodating 20 crucibles. Prior to analysis, all samples were oven-dried at 105 °C and subsequently exposed to thermochemical treatments defined by six target temperatures (180, 190, 200, 210, 220, and 230 °C) and three residence times (10, 15, and 20 min) across five selected wood species (Supplementary Table S2). A total of 108 samples were analyzed, allocated across 18 crucibles, as shown in Supplementary Figure S5.

### 5.1. Experimental Design

A factorial experimental design was established using the Design of Experiments (DOE) module in Statistica 12. A central composite design (CCD) incorporating two independent variables—temperature and time—was applied to assess their influence on the responses of interest (Supplementary Table S2). The design incorporated three center points to generate replicated measurements under identical conditions, thereby improving the reliability of the experimental error estimate.

### 5.2. Preparation of Solutions

Alcoholic solutions were prepared using 96% (*v*/*v*) grain alcohol, which was diluted to final concentrations of 5% (*v*/*v*) and 38% (*v*/*v*). For each solution, 3 g of thermally treated wood were added to 500 mL and the mixtures were stored for 14 days at room temperature. After this extraction period, the wood samples were removed, and the resulting solutions were submitted to sensory analysis.

### 5.3. Sensory Analysis

Sensory analyses were conducted at the University of Brasília (UnB). The sensory panel was composed of trained jurors who underwent both theoretical and practical training sessions using an off-flavor kit (FlavorActiv, Oxfordshire, UK), administered by qualified personnel. Following this training and evaluation process, a sensory panel was established at UnB and was responsible for assessing the samples analyzed in the present study.

Each sample was evaluated across two sensory categories: aroma and flavor. The evaluation form included predefined descriptors typically associated with wood-aged beverages, along with an open field allowing panelists to report any additional perceived notes. Scores were assigned on a scale from 1 (lowest perception) to 5 (highest perception).

The results of the sensory analyses regarding aromas and flavors are presented in spider graphs for each wood species. For data processing, the arithmetic mean of the evaluators’ scores for each sensory attribute was calculated, and the resulting average profiles were integrated into the model construction.

### 5.4. Five-Wood Cachaça Production

Following the sensory evaluations, barrels were produced from each selected wood species using the optimized toasting conditions identified to enhance the desired characteristics. Each barrel was filled with 10 L of *cachaça* (38% *v*/*v* alcohol) and aged for one year. Upon completion of the aging period, the spirits were blended in equal proportions, and the resulting five-wood *cachaça* was subjected to physicochemical analysis.

### 5.5. Physicochemical Analysis of the Final Five-Wood Cachaça

The physicochemical characterization of the five-wood *cachaça* encompassed the determination of density, volatile acidity, copper content, and volatile and contaminant compounds. Density was measured by immersing a densimeter in a graduated cylinder containing the sample and recording the reading at the liquid meniscus.

Volatile acidity was determined following the Adolfo Lutz Institute methodology (Instituto Adolfo Lutz, 2008) [30], based on the difference between total and fixed acidity. For this analysis, 50 mL of each sample was titrated with 0.01 mol·L^−1^ sodium hydroxide (NaOH), using phenolphthalein as an indicator, until the persistence of a faint pink coloration.

Copper content was quantified via classical titration using 0.01 mol·L^−1^ sodium thiosulfate, previously standardized with potassium dichromate (K_2_Cr_2_O_7_), until the solution reached a pale-yellow color (Da Silva et al., 2021) [23]. A 1.0% starch suspension was then added, and titration was continued to the colorless endpoint. Copper concentration (mg Cu^2+^·L^−1^) was calculated using Equation (3):

mg Cu^2+^·L^−1^ = (Vg × M × 63.5 × 1000)/Va (3)

where Vg is the titrant volume (L), M is the molarity of sodium thiosulfate (mol·L^−1^), 63.5 is the atomic mass of copper (g·mol^−1^), 1000 is a unit conversion factor, and Va is the volume of the standard solution (L).

Volatile compounds were analyzed via gas chromatography coupled to mass spectrometry (GC–MS) using a GC-2010 system equipped with a GCMS-QP2010 Plus detector and an AOC-5000 injector (Shimadzu Corporation, Kyoto, Japan). Sample preparation consisted of centrifuging 150 g of alcoholic solution at 2000 rpm for 5 min, extracting it with 75 mL of dichloromethane in a separatory funnel, shaking, and allowing the phases to separate for 2 h. The organic phase was collected and centrifuged again for 4 min at 2000 rpm. Analytical conditions included electron ionization at 70 eV, injection of 1 μL of sample at 200 °C with a 1:10 split ratio, and use of a Restek Rtx-5MS column (30 m × 250 μm × 0.25 μm) (Restek Corporation, Bellefonte, PA, USA). Helium served as the carrier gas at 1.4 mL/min, with a scan range of 30–450 *m*/*z*. The oven temperature program initiated at 50 °C for 1 min, followed by a ramp from 50 to 180 °C at a rate 2 °C/min.

## 6. Conclusions

This study demonstrates that Brazilian wood species (Amburana, Balsam, Ipê, Jequitibá, and Jaqueira) exhibit distinct physicochemical and sensory properties that can be strategically exploited for *cachaça* maturation. Variations in extractives, lignin, and holocellulose contents were closely associated with the development of specific aroma and flavor profiles, with some species showing sensory intensities comparable to or exceeding those of oak. An AI-assisted predictive model integrating compositional data, toasting parameters, sensory descriptors, and literature evidence was developed to optimize wood-toasting conditions and exhibited satisfactory statistical performance, explaining approximately 67.4% of the total variance. The practical applicability of this approach was validated through the production of a five-wood *cachaça* that met physicochemical quality standards and regulatory requirements. These findings highlight the potential of AI-guided, multi-criteria optimization as a rational strategy for flavor design in *cachaça* production, supporting sensory diversification and reduced reliance on oak. Nevertheless, the limited aging period and sensory panel size indicate the need for future studies involving extended maturation, broader model validation, and expanded sensory evaluation.

## Figures and Tables

**Table 1:** Extractives and moisture contents of the analyzed wood samples.

Sample	Extractives (%)	Humidity (%)
Amburana	7.05	5.12
Balsam	16.57	5.20
Oak *	16.55	5.50
Jaqueira	7.95	4.34
Ipê	28.19	5.33
Jequitibá	7.71	5.24

* Standard wood traditionally employed for aging.

**Table 2:** Ash (without extractives), lignin, and holocellulose contents of the analyzed wood samples.

Sample	Ash (%)	Lignin (%)	Holocellulose (%)
Amburana	0.84	43.78	56.07
Balsam	0.75	44.32	54.93
Oak *	0.14	40.39	59.53
Jaqueira	0.25	50.14	49.62
Ipê	0.14	46.89	52.97
Jequitibá	0.62	43.12	56.26

* Standard wood traditionally employed for aging.

**Table 3:** Wood species, associated aroma and flavor characteristics, and related compounds or observations.

Wood Species	Aroma Characteristics	Flavor (Taste) Characteristics	Associated Compounds/Observations
Jequitibá	Pronounced vanilla aroma; malt notes also perceived	Resinous mouthfeel	Vanilla flavor extracted from the wood. Resinous sensation associated with gallic acid derived from lignina, spiced notes linked to lignin-derived compounds such as syringaldehyde, sinapaldehyde, and coniferaldehydes.
Jaqueira	Malt, vanilla, and predominantly spiced aromas	Burnt flavor with tannic sensation; presence of spiced notes	Burnt flavor attributed to the toasting of the wood, which enhanced tannic characteristics. Spiced flavor related to compounds associated with this flavor profile.
Balsam	Evident vanilla and fruity aromas	Vanilla and fruity flavors highlighted	Vanilla associated with lignin-derived compounds such as vanillin; fruity notes linked to catechin and scopoletin, also derived from lignin.
Carvalho (Oak)	Burnt, resinous, vanilla, spiced, and malt aromas	Resinous, tannic, and spiced flavors	Exhibited the broadest range of flavors, with more pronounced intensities, particularly in taste.
Amburana	Highly intense spiced aroma	Extremely intense spiced flavor	Showed the highest sensory intensities among all samples, with intensity for spiced aroma and spiced flavor.
Ipê	Burnt, resinous, and vanilla aromas	Resinous, tannic, and spiced flavors	Displayed a broad flavor profile with stronger intensities in taste, similar to oak.

**Table 4:** Conditions suggested by the sensory analyses and the respective intensities of aromas and flavors.

Wood Sample	Temperatura (°C)	Time (min)	Aroma	Flavor
Amburana	190	15	1.41	1.75
Balsam	220	20	0.93	1.17
Oak	230	20	1.37	1.47
Jaqueira	180	20	1.26	1.37
Ipê	230	10	1.62	1.58
Jequitibá	180	15	1.07	1.43

**Table 5:** ANOVA of the quadratic model assessing the effects of temperature and time on wood-toasting intensity.

	Source of Variation	SS	GL	MQ	Fcal	Ftab
SG *	Regression	62.28	2	31.14	9.31	4.46
	Lack of adjustment	30.10	7	4.30	295.92	19.33
	Pure error	0.317	2	1.4 × 10^−2^		
	Total	92.38	10			
% of variance explained (R^2^) 67.41						
Max. % of variance explained (R^2^_max_.) 99.96						

* Note: SG indicates that the regression is significant. SS refers to the sum of squares, GL to the degrees of freedom, and MQ to the mean square. Fcal and Ftab represent the calculated and tabulated F values, respectively.

**Table 6:** Parameters and analytes determined by standard methods in the final five-wood *cachaça*.

Parameter or Analyte	Experimental Measurement	Unit of Measurement
Relative density (20 °C)	0.95 ± 0.0	— (unitless ratio)
Copper (Cu^3+^)	1.09 ± 0.07	mg L^−1^ or ppm
Total dry extract	0.68 ± 0.01	g L^−1^
Actual alcoholic strength	39.30 ± 0.03	°GL
Volatile acidity	79.29 ± 0.06	mg L^−1^
Total higher alcohols	248.56 ± 2.36	mg L^−1^
n-Propyl alcohol	57.23 ± 1.00	mg L^−1^
Isobutyl alcohol	69.24 ± 0.41	mg L^−1^
Isoamyl alcohol	122.08 ± 0.81	mg L^−1^
sec-Butyl alcohol	(LOD: 0.48/LOQ: 1.60) < LOD	mg L^−1^
n-Butyl alcohol	(LOD: 0.36/LOQ: 1.22) < LOD	mg L^−1^
Furfural	6.13 ± 0.08	mg L^−1^
Aldehydes	14.31 ± 0.07	mg L^−1^
Esters	46.51 ± 0.03	mg L^−1^
Methyl alcohol (Methanol)	(LOD: 0.19/LOQ: 0.63) 5.77 ± 0.07	mg L^−1^
Acrolein	(LOD: 0.08/LOQ: 0.25) < LOQ	μg L^−1^
Ethyl carbamate	209.04 ± 0.89	μg L^−1^

LOD: Limit of Detection. LOQ: Limit of Quantification.

**Table 7:** Wood species used in this study.

Wood Common Name	Scientific Name
Oak *	*Quercus* spp.
Amburana	*Amburana cearenses*
Balsam	*Myroxylon balsamum*
Ipê	*Tabebuia* spp.
Jequitibá	*Cariniana* spp.
Jaqueira	*Artocarpus heterophyllus*

* Standard wood traditionally used in maturation processes.

## Data Availability

The original contributions presented in this study are included in the article/Supplementary Material. Further inquiries can be directed to the corresponding author.

## Supplementary Materials

The following supporting information can be downloaded at: https://www.mdpi.com/article/10.3390/molecules31040633/s1. Figure S1: Schematic representation of spirit maturation using reused barrels, virgin barrels, or the addition of wood pieces to impart flavor. Figure S2: Key compounds in oak extracts and the structural constituents of the oak cell wall, compiled from published literature data. Figure S3: Spider charts of the samples profiles: Jequitibá (a) aroma and (b) flavor; Jaqueira (c) aroma and (d) flavor; Balsam: (e) aroma and (f) flavor; Oak (g) aroma and (h) flavor; Amburana (i) aroma and (j) flavor; Ipê (aroma) and (l) flavor. Figure S4: Experimental toasting system: (1) N_2_ cylinder, (2) gas control rotameter, (3) MACRO TGA-2000 thermogravimeter, and (4) computer. Figure S5: Interior of the MACRO TGA-2000 system and the support structure used to hold the wood samples. Samples S1–S6 include all analyzed specimens, with oak serving as the reference. Table S1: Key wood-derived compounds and their contribution to sensory characteristics. Table S2: Experimental design.
