# Supplementary material for: Optimizing the Flavor Profile of Brazilian Spirits: Torrefaction Modeling of Native Woods for Cachaça Maturation
Source: Molecules. 2026 Feb 12;31(4):633. doi: 10.3390/molecules31040633 (PMC12943465; doi:10.3390/molecules31040633)
Supplement: Supplementary file 1 [file molecules-31-00633-s001.zip › molecules-4115576-supplementary.pdf]

Figure caption

Figure S1. Schematic representation of spirit maturation using reused barrels, virgin barrels, or the addition of wood pieces to impart flavor.

Figure S2. Key compounds in oak extracts and the structural constituents of the oak cell wall, compiled from published literature data.

Figure S3. Spider charts of the samples profiles: Jequitibá (a) aroma and (b) flavor; Jaqueira (c) aroma and (d) flavor; Balsam: (e) aroma and (f) flavor; Oak (g) aroma and (h) flavor; Amburana (i) aroma and (j) flavor; Ipê (aroma) and (l) flavor.

Figure S4. Experimental toasting system: (1) N_2 cylinder, (2) gas control rotameter, (3) MACRO TGA-2000 thermogravimeter, and (4) computer.

Figure S5. Interior of the MACRO TGA-2000 system and the support structure used to hold the wood samples. Samples S1–S6 include all analyzed specimens, with oak serving as the reference.

Table caption

Table S1. Key wood-derived compounds and their contribution to sensory characteristics.

Table S2. Experimental design.

Figure S1

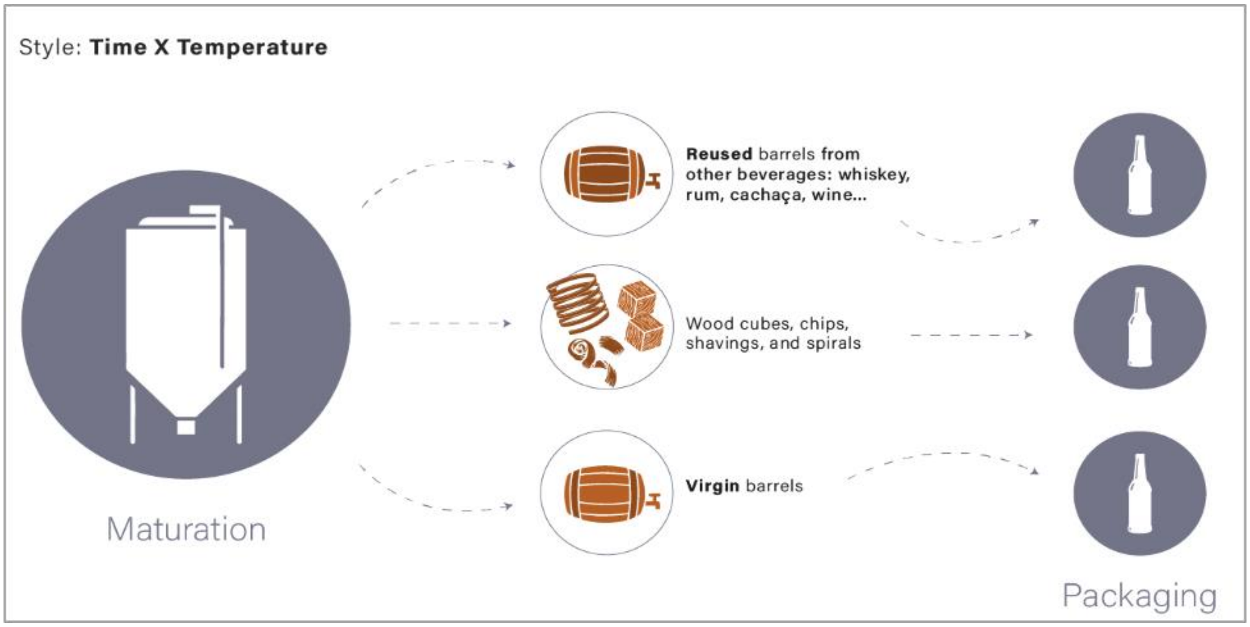

Figure S2

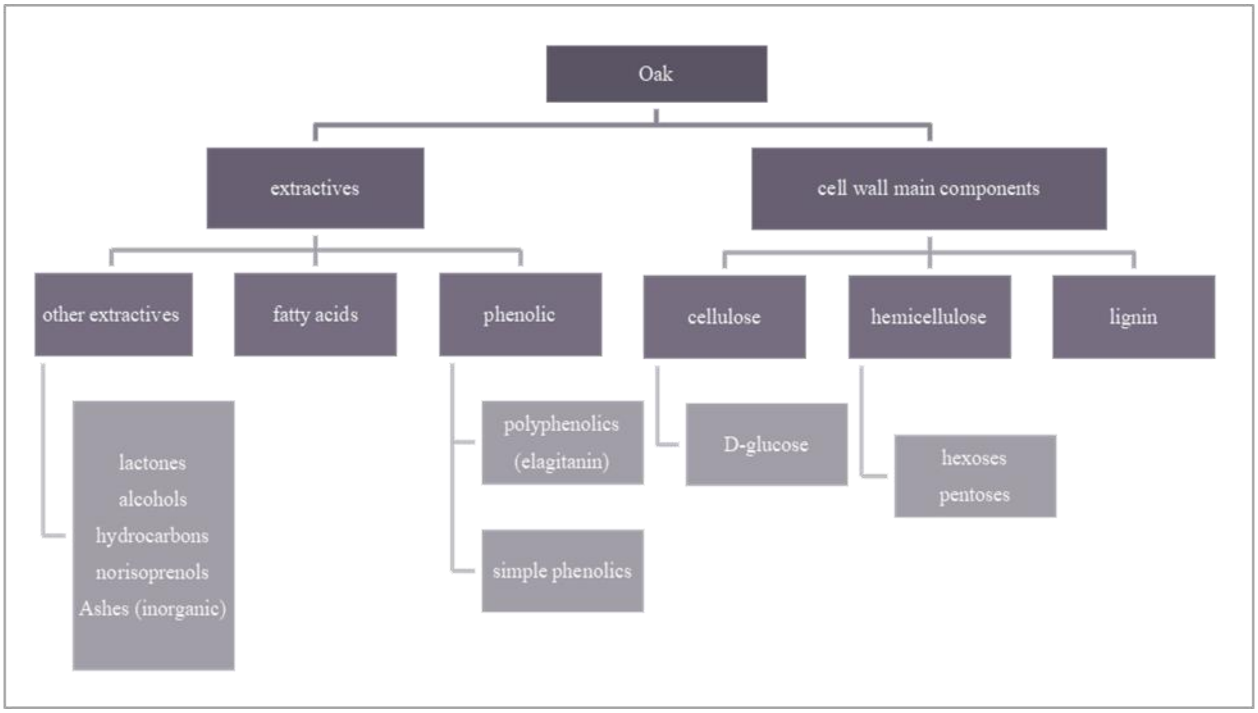

Figure S3

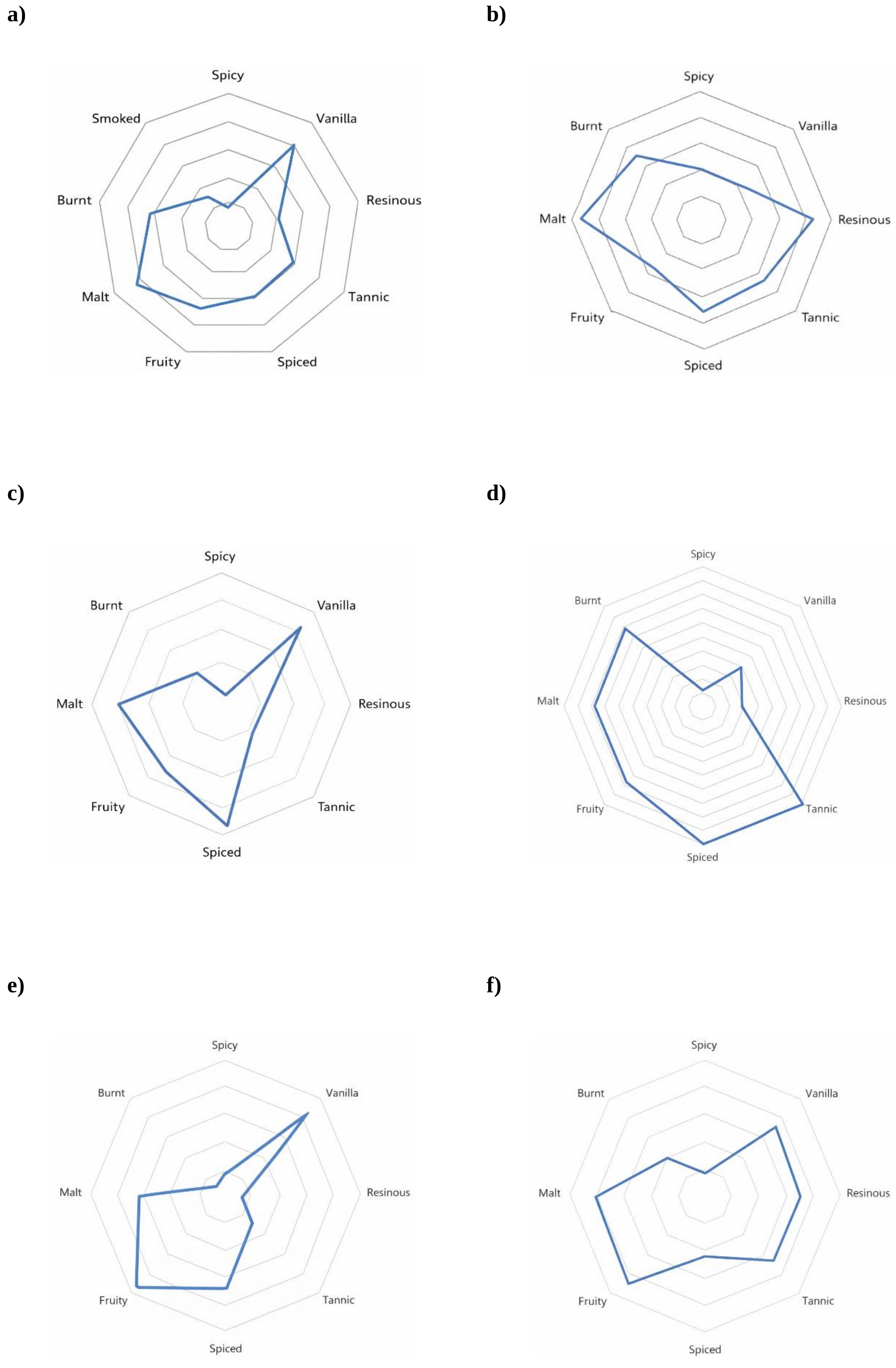
a)
Spicy
Vanilla
Resinous
Tannic
Spiced
Fruity
Malt
Burnt
Smoked
b)
Spicy
Vanilla
Resinous
Tannic
Spiced
Fruity
Malt
Burnt
c)
Spicy
Vanilla
Resinous
Tannic
Spiced
Fruity
Malt
Burnt
d)
Spicy
Vanilla
Resinous
Tannic
Spiced
Fruity
Malt
Burnt
e)
Spicy
Vanilla
Resinous
Tannic
Spiced
Fruity
Malt
Burnt
f)
Spicy
Vanilla
Resinous
Tannic
Spiced
Fruity
Malt
Burnt

g)

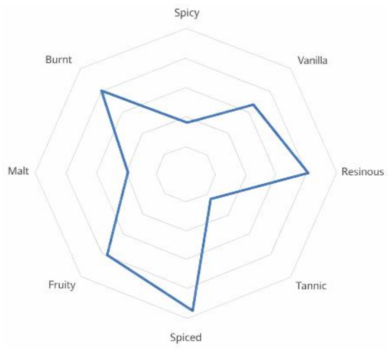
Spicy
Vanilla
Resinous
Tannic
Spiced
Fruity
Malt
Burnt

h)

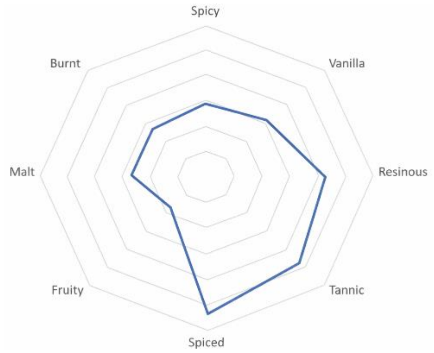
Spicy
Vanilla
Resinous
Tannic
Spiced
Fruity
Malt
Burnt

i)

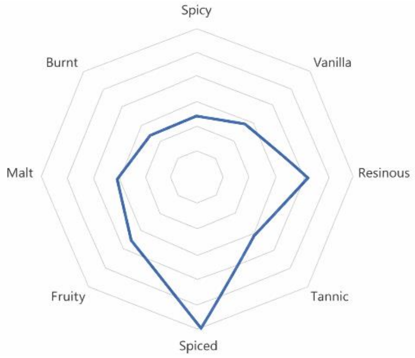
Spicy
Vanilla
Resinous
Tannic
Spiced
Fruity
Malt
Burnt

j)

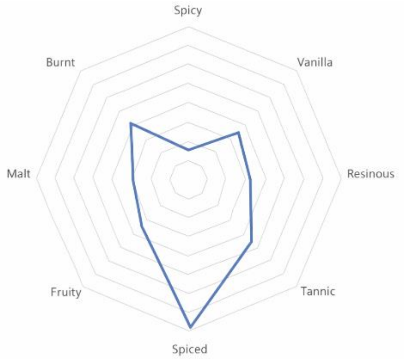
Spicy
Vanilla
Resinous
Tannic
Spiced
Fruity
Malt
Burnt

k)

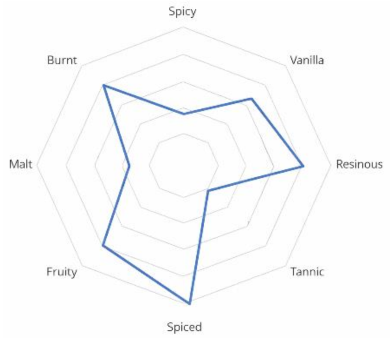
Spicy
Vanilla
Resinous
Tannic
Spiced
Fruity
Malt
Burnt

l)

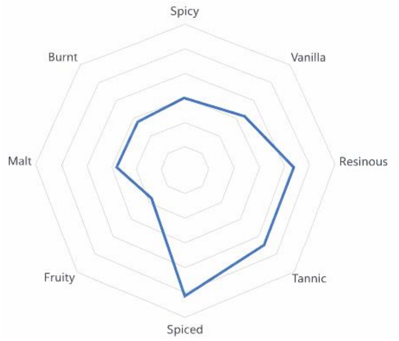
Spicy
Vanilla
Resinous
Tannic
Spiced
Fruity
Malt
Burnt

Figure S4

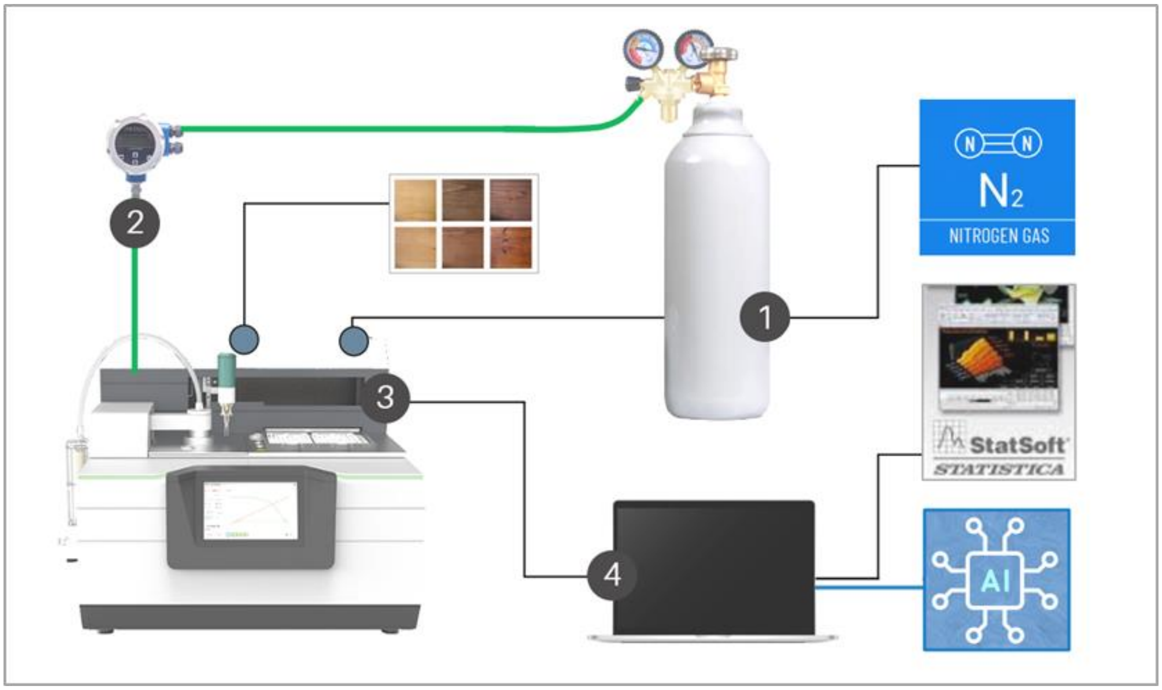

Fig. S5

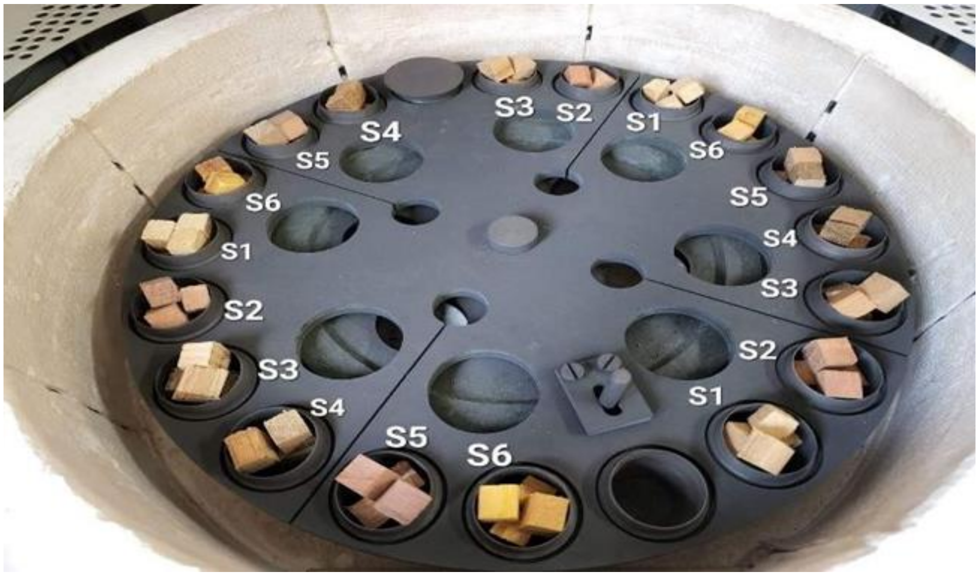

Table S1

Name	Chemical compound	Chemical structure	Associated flavor and aroma	Occurrence
**Vanillin**	3-methoxy-4-hydroxybenzaldehyde	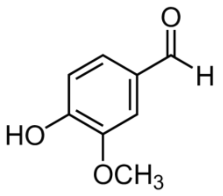	Vanilla	Jequitibá, Amburana, Balsam, Castanheira and Oak
**Gallic Acid**	3,4,5-trihydroxybenzoic acid	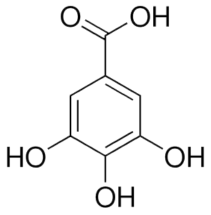	Vegetable and resinous aromas	Jequitibá, Amburana, Balsam, Castanheira and Oak
**Vanillic acid**	4-hydroxy-3-methoxybenzoic acid	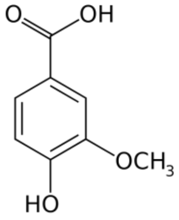	Vanilla	Jequitibá, Amburana, Balsam, Castanheira and Oak
**Syringic acid**	3,4,5-trimethyloxybenzoic acid	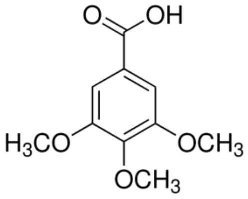	Peppery sensations and spicy aromas	Jequitibá, Amburana, Balsam, Castanheira and Oak
**Syringald ehyde**	4-Hydroxy-3,5-dimethoxybenzaldehyde	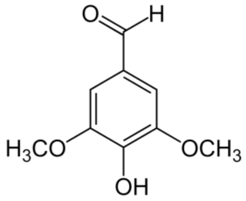	Peppery sensations and spicy aromas	Jequitibá, Amburana, Balsam, Castanheira and Oak

**Coniferyl aldehyde**	4-hydroxy-3-methoxycinnamaldehyde	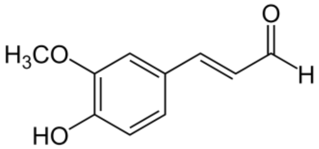	Peppery sensations and spicy aromas	Jequitibá, Amburana, Balsam, Castanheira and Oak
**Sinapaldehyde**	3,5-Dimethoxy- 4-hydroxycinnam aldehyde	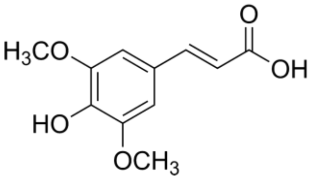	Peppery sensations and spicy aromas	Jequitibá, Amburana, Balsam, Castanheira and Oak
**Sinapic acid**	3-(4-hydroxy-3,5-dimethoxyphenyl)-prop-2-enoic acid	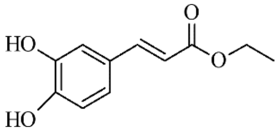	Peppery sensations and spicy aromas	Jequitibá, Amburana, Balsam, Castanheira and Oak
**Ellagic acid**	2,3,7,8-tetrahydroxychromeno[5,4,3- cde]chromene- 5,10-dione	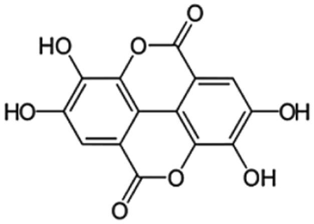	Acidity, bitterness	Castanheira and Oak
**P-coumaric acid**	(E)-3-(4-hydroxyphenyl)-2-propenoic acid	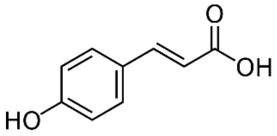	Vanilla	Jequitibá, Amburana, Balsam, Castanheira and Oak
**Coumarin**	2*H*-1-benzopirano-2-ona	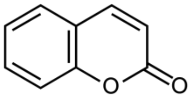	Vanilla	Jequitibá, Amburana, Balsam, Castanheira and Oak

**Capric Acid**	Decanoic acid	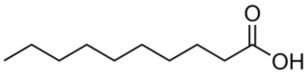	Odor characteristic of goats	Jequitibá
**Liquiritigenin**	(2S)-7-hydroxi-2-(4-hydroxyphenyl)- 2,3-dihydro-4H-cromen-4-one	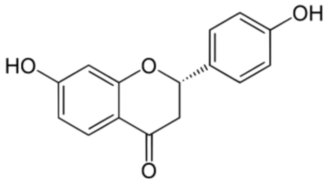		Jequitibá
**Eugenol**	4-Allyl-2-methoxyphenol	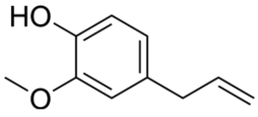	Produced by the breakdown of lignin when the wood is toasted and contributes to the spicy character, such as cloves and smoke.	Jequitibá, Amburana, Balsam
**Caprylic acid**	Octanoic acid	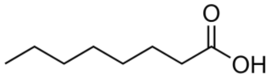	Aroma of tallow, wax or soap	Jequitibá
**Naringenin**	5,7-dihydroxy-2- (4-hydroxyphenyl) chroman-4-one	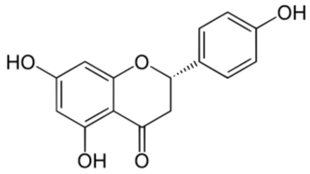	Characterist ic bitter taste of grapefruit juice	Amburana
**Pectolinar ingenin**	5,7-dihydroxy- 6-methoxy-2- (4-methoxyphenyl)	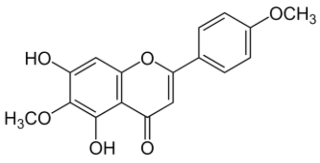	Characterist ic bitter taste of grapefruit juice	Amburana

**Catechin**	(2R,3S)-2-(3,4-dihydroxypheny l)-3,4-dihydro- 2H-chromene-3,5,7-triol	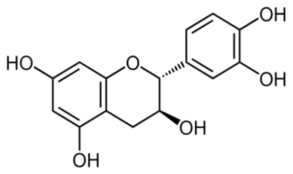	Bitter taste of green tea	Amburana, Balsam and Oak
**4-Methylum belliferone**	7-Hydroxy-4-methylcoumarin	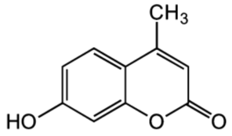		Amburana
**Genistein**	5,7-dihydroxy-3-(4-hydroxyphenyl)-4H-chromen-4-one	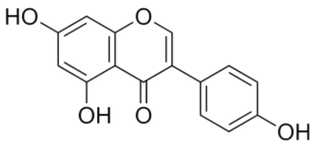	Bitterness and astringency	Balsam
**Biochanin**	5,7-dihydroxy- 3- (4-methoxyphenyl)chromen-4-one	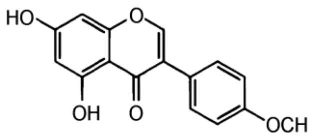		Balsam
**Scopoletin**	7-hydroxy-6-methoxychrome n-2-one	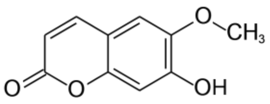	Fruity	Balsam
**Ethyl gallate**	Ethyl 3,4,5-trihydroxybenzoate	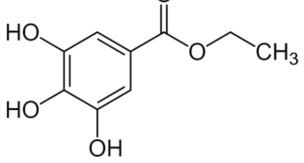		Castanheira

**4-methylumbelliferone**	4-methylumbelliferone	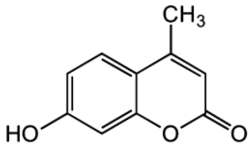		Castanheira
**5 - HMF**	5-hydroxymethylf urfural (5-HMF)	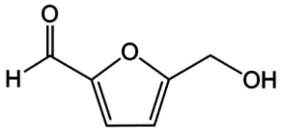	Toast, caramel, bread and almonds give a characteristic brown color	Oak
**Guaiacol**	2-methoxyphenol	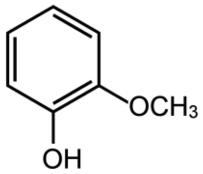	A smoky flavor is characteristic of compounds containing guaiacol.	Jequitibá, Amburana, Balsam, Castanheira and Oak
**Furfural**	furan-2- carboxyaldehyde	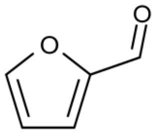	It smells like almonds. A derivative of furfural is furfuryl-mercaptan, which gives roasted coffee its aroma. It imparts a caramel, peanut butter aroma	Oak

Table S2

Amburana						
Sample	Treatment Content (%)	Temp (°C)	Time (min)	pH		
				pH 1	pH 2	pH 3
A1	5	180	10	5.35	5.35	5.35
A2	5	180	15	5.15	5.14	5.13
A3	5	180	20	5.18	5.19	5.18
A4	5	190	10	5.05	5.04	5.04
A5	5	190	15	5.0	4.97	4.97
A6	5	190	20	5.38	5.41	5.4
A7	5	200	10	5.3	5.31	5.32
A8	5	200	15	4.84	4.87	4.88
A9	5	200	20	4.92	4.91	4.94
A10	5	210	10	5.85	5.87	5.88
A11	5	210	15	4.91	4.91	4.94
A12	5	210	20	5.27	5.27	5.29
A13	5	220	10	5.48	5.5	5.49
A14	5	220	15	5.99	6.01	5.99
A15	5	220	20	5.12	5.1	5.08
A16	5	230	10	4.84	4.85	4.83
A17	5	230	15	4.96	4.97	4.94
A18	5	230	20	4.94	4.96	4.92
A19	38	180	10	4.8	4.79	4.75
A20	38	180	15	4.81	4.77	4.8
A21	38	180	20	4.98	4.96	5.0
A22	38	190	10	4.94	4.88	4.92
A23	38	190	15	5.0	5.02	5.04
A24	38	190	20	4.94	4.92	4.97
A25	38	200	10	5.09	5.14	5.1
A26	38	200	15	4.91	4.96	4.96
A27	38	200	20	5.0	4.94	4.96
A28	38	210	10	4.85	4.87	4.85
A29	38	210	15	5.0	5.02	4.99
A30	38	210	20	5.0	5.0	5.02
A31	38	220	10	4.82	4.83	4.81
A32	38	220	15	4.9	4.94	4.89
A33	38	220	20	4.67	4.71	4.69
A34	38	230	10	4.86	4.91	4.88
A35	38	230	15	4.9	4.89	4.94
A36	38	230	20	4.92	4.94	4.91

Balsam						
Sample	Treatment			pH		
	Content (%)	Temp (°C)	Time (min)	pH 1	pH 2	pH 3
B1	5	180	10	4.6	4.46	4.55
B2	5	180	15	5.15	5.25	5.28
B3	5	180	20	5.16	5.25	5.25
B4	5	190	10	5.54	5.65	5.7
B5	5	190	15	5.57	5.6	5.75
B6	5	190	20	5.52	5.53	5.5
B7	5	200	10	5.33	5.37	5.35
B8	5	200	15	4.1	4.12	4.08
B9	5	200	20	4.25	4.27	4.3
B10	5	210	10	5.9	6.07	6.07
B11	5	210	15	5.7	5.72	5.8
B12	5	210	20	4.38	4.42	4.4
B13	5	220	10	4.67	4.7	4.71
B14	5	220	15	5.5	5.65	5.7
B15	5	220	20	4.85	4.87	4.85
B16	5	230	10	5.6	5.65	5.7
B17	5	230	15	5.4	5.39	5.46
B18	5	230	20	4.52	4.52	4.56
B19	38	180	10	4.96	4.94	4.9
B20	38	180	15	4.6	4.59	4.57
B21	38	180	20	4.77	4.8	4.8
B22	38	190	10	4.96	4.94	4.97
B23	38	190	15	5.1	5.13	5.15
B24	38	190	20	5.07	5.06	5.09
B25	38	200	10	5.2	5.22	5.27
B26	38	200	15	4.92	4.99	5.0
B27	38	200	20	4.9	4.94	4.9
B28	38	210	10	4.85	4.84	4.82
B29	38	210	15	4.85	4.81	4.83
B30	38	210	20	4.94	4.92	5.02
B31	38	220	10	4.72	4.79	4.77
B32	38	220	15	4.85	4.87	4.87
B33	38	220	20	4.97	5.0	5.02
B34	38	230	10	4.72	4.79	4.7
B35	38	230	15	4.9	4.87	4.92
B36	38	230	20	4.85	4.84	4.85

Oak						
Sample	Treatment Content (%)	Temp (°C)	Time (min)	pH		
				pH 1	pH 2	pH 3
C1	5	180	10	4.2	4.18	4.15
C2	5	180	15	4.0	3.95	3.93
C3	5	180	20	3.8	3.82	3.85
C4	5	190	10	4.06	4.0	4.0
C5	5	190	15	3.92	3.95	3.95
C6	5	190	20	3.79	3.81	3.84
C7	5	200	10	3.88	4.0	3.92
C8	5	200	15	3.79	3.78	3.8
C9	5	200	20	4.05	4.1	4.1
C10	5	210	10	4.55	4.52	4.48
C11	5	210	15	4.22	4.14	4.17
C12	5	210	20	4.0	4.0	4.0
C13	5	220	10	3.8	3.87	3.9
C14	5	220	15	3.77	3.88	3.84
C15	5	220	20	4.05	4.0	4.02
C16	5	230	10	3.9	3.92	3.95
C17	5	230	15	4.0	4.02	4.05
C18	5	230	20	4.0	4.08	4.09
C19	38	180	10	4.0	4.05	4.04
C20	38	180	15	3.77	3.78	3.75
C21	38	180	20	3.7	3.75	3.75
C22	38	190	10	3.89	3.95	3.99
C23	38	190	15	3.95	3.97	3.95
C24	38	190	20	4.12	4.14	4.1
C25	38	200	10	4.1	4.2	4.17
C26	38	200	15	4.0	3.94	4.0
C27	38	200	20	4.25	4.24	4.3
C28	38	210	10	3.81	3.86	3.94
C29	38	210	15	4.2	4.17	4.19
C30	38	210	20	3.95	3.97	3.99
C31	38	220	10	3.81	3.8	3.8
C32	38	220	15	3.8	3.85	3.85
C33	38	220	20	3.9	3.95	3.95
C34	38	230	10	3.91	3.92	3.94
C35	38	230	15	3.81	3.84	3.86
C36	38	230	20	3.97	4.0	4.05

Ipê						
Sample	Treatment			pH		
	Content (%)	Temp (°C)	Time (min)	pH 1	pH 2	pH 3
I1	5	180	10	6.07	6.04	6.1
I2	5	180	15	6.26	6.24	6.23
I3	5	180	20	6.44	6.4	6.46
I4	5	190	10	6.64	6.68	6.62
I5	5	190	15	6.54	6.59	6.56
I6	5	190	20	6.55	6.51	6.51
I7	5	200	10	6.54	6.53	6.58
I8	5	200	15	6.32	6.35	6.34
I9	5	200	20	5.97	5.95	5.92
I10	5	210	10	6.46	6.55	6.6
I11	5	210	15	4.44	4.42	4.38
I12	5	210	20	5.77	5.97	5.87
I13	5	220	10	6.4	6.7	6.71
I14	5	220	15	6.5	6.57	6.5
I15	5	220	20	6.63	6.65	6.74
I16	5	230	10	6.75	6.78	6.72
I17	5	230	15	5.4	5.22	5.13
I18	5	230	20	6.0	6.08	6.1
I19	38	180	10	5.54	5.4	5.4
I20	38	180	15	4.92	4.9	4.94
I21	38	180	20	5.1	5.05	5.02
I22	38	190	10	5.96	5.95	5.96
I23	38	190	15	5.6	5.69	5.71
I24	38	190	20	6.1	6.32	6.5
I25	38	200	10	5.6	5.54	5.55
I26	38	200	15	5.15	5.25	5.16
I27	38	200	20	5.57	5.68	5.72
I28	38	210	10	5.3	5.35	5.35
I29	38	210	15	5.5	5.59	5.59
I30	38	210	20	5.85	6.0	6.05
I31	38	220	10	5.35	5.37	5.35
I32	38	220	15	5.57	5.69	5.67
I33	38	220	20	5.51	5.57	5.47
I34	38	230	10	5.3	5.38	5.4
I35	38	230	15	6.0	6.18	6.18
I36	38	230	20	5.75	5.6	5.6

Jequitibá						
Sample	Treatment			pH		
	Content (%)	Temp (°C)	Time (min)	pH 1	pH 2	pH 3
J1	5	180	10	5.16	5.12	5.09
J2	5	180	15	5.17	5.21	5.25
J3	5	180	20	5.29	5.32	5.34
J4	5	190	10	4.27	4.25	4.24
J5	5	190	15	4.89	4.88	4.9
J6	5	190	20	4.73	4.72	4.7
J7	5	200	10	5.83	5.75	5.8
J8	5	200	15	4.34	4.3	4.28
J9	5	200	20	4.54	4.52	4.52
J10	5	210	10	5.58	5.66	5.66
J11	5	210	15	4.7	4.8	4.75
J12	5	210	20	4.59	4.55	4.54
J13	5	220	10	4.86	4.85	4.89
J14	5	220	15	5.65	5.68	5.72
J15	5	220	20	4.75	4.7	4.68
J16	5	230	10	4.8	4.8	4.81
J17	5	230	15	4.7	4.7	4.71
J18	5	230	20	5.43	5.49	5.48
J19	38	180	10	4.7	4.68	4.69
J20	38	180	15	4.76	4.72	4.71
J21	38	180	20	4.8	4.84	4.87
J22	38	190	10	5.13	5.18	5.2
J23	38	190	15	5.35	5.4	5.38
J24	38	190	20	4.56	4.53	4.52
J25	38	200	10	4.67	4.65	4.72
J26	38	200	15	5.12	5.21	5.17
J27	38	200	20	4.83	4.79	4.78
J28	38	210	10	4.77	4.75	4.74
J29	38	210	15	4.88	4.86	4.88
J30	38	210	20	5.0	4.96	4.95
J31	38	220	10	4.92	4.88	4.95
J32	38	220	15	4.77	4.8	4.8
J33	38	220	20	4.52	4.49	4.49
J34	38	230	10	4.86	4.85	4.82
J35	38	230	15	4.85	4.86	4.83
J36	38	230	20	4.76	4.7	4.74

Jaqueira						
Sample	Treatment Content (%)	Temp (°C)	Time (min)	pH<br>pH 1	pH 2	pH 3
Q1	5	180	10	5.22	5.16	5.14
Q2	5	180	15	5.15	5.14	5.14
Q3	5	180	20	5.12	5.11	5.11
Q4	5	190	10	5.22	5.2	5.22
Q5	5	190	15	5.11	5.08	5.08
Q6	5	190	20	4.7	4.66	4.66
Q7	5	200	10	5.03	5.03	5.03
Q8	5	200	15	5.11	5.08	5.08
Q9	5	200	20	4.43	4.38	4.37
Q10	5	210	10	5.11	4.95	4.89
Q11	5	210	15	5.01	4.98	4.96
Q12	5	210	20	4.46	4.43	4.4
Q13	5	220	10	4.39	4.35	4.36
Q14	5	220	15	4.61	4.61	4.6
Q15	5	220	20	5.19	5.19	5.2
Q16	5	230	10	4.27	4.25	4.25
Q17	5	230	15	5.34	5.32	5.35
Q18	5	230	20	4.72	4.69	4.69
Q19	38	180	10	4.71	4.79	4.75
Q20	38	180	15	4.95	4.93	4.85
Q21	38	180	20	4.78	4.82	4.77
Q22	38	190	10	5.15	5.22	5.24
Q23	38	190	15	4.98	4.9	4.97
Q24	38	190	20	4.79	4.8	4.85
Q25	38	200	10	4.89	4.88	4.86
Q26	38	200	15	5.35	5.12	5.04
Q27	38	200	20	5.01	4.94	4.95
Q28	38	210	10	4.98	4.96	4.95
Q29	38	210	15	4.94	4.87	4.89
Q30	38	210	20	4.83	4.84	4.79
Q31	38	220	10	4.87	4.88	4.84
Q32	38	220	15	4.84	4.84	4.89
Q33	38	220	20	4.88	4.88	4.84
Q34	38	230	10	4.92	4.83	4.83
Q35	38	230	15	4.86	4.84	4.82
Q36	38	230	20	4.95	4.88	4.89
